# Development of an online educational toolkit for sexual orientation and gender identity minority nursing care*

**DOI:** 10.1590/1518-8345.4712.3470

**Published:** 2021-08-30

**Authors:** Erin Ziegler, Marian Luctkar-Flude, Benjamin Carroll, Jane Tyerman, Lillian Chumbley, Chris Shortall

**Affiliations:** 1 Ryerson University, Daphne Cockwell School of Nursing, Toronto, ON, Canada.; 2 Queen’s University, School of Nursing, Kingston, ON, Canada.; 3 University of Ottawa, School of Nursing, Ottawa, ON, Canada.; 4 Trent University, External Relations & Advancemente, Peterborough, ON, Canada.; 5 Rainbow Health Consulting Ltd, St. John’s, Newfoundland and Labrador, Canada.

**Keywords:** Sexual and Gender Minorities, Patient Simulation, Culturally Competent Care, Nursing, Education, Minorias Sexuais e de Gênero, Simulação de Paciente, Assistência à Saúde Culturalmente Competente, Enfermagem, Educação, Minorias Sexuales y de Género, Simulación de Paciente, Asistencia Sanitaria Culturalmente Competente, Enfermería, Educación

## Abstract

**Objective:**

to develop and implement an online education resources to address a gap in nursing education regarding the concept of cultural humility and its application to healthcare encounters with persons who identify as lesbian, gay, bisexual, transgender, queer, intersex (LGBTQI) or Two-Spirit. Improved understanding of LGBTQI and Two-Spirit community health issues is essential to reducing the healthcare access barriers they currently face.

**Method:**

an online educational toolkit was developed that included virtual simulation games and curated resources. The development process included community involvement, a team-building meeting, development of learning outcomes, decision-point maps and scriptwriting for filming. A website and learning management system was designed to present learning objectives, curated resources, and the virtual games.

**Results:**

the *Sexual Orientation and Gender Identity Nursing Toolkit* was created to advance cultural humility in nursing practice. The learning toolkit focuses on encounters using cultural humility to meet the unique needs of LGBTQI and Two-Spirit communities.

**Conclusion:**

our innovative online educational toolkit can be used to provide professional development of nurses and other healthcare practitioners to care for LGBTQI and Two-Spirit individuals.

## Abstract (pt)

**Objetivo:**

desenvolver e implementar uma ferramenta educacional online para atender a uma demanda na educação de enfermagem a respeito do conceito de humildade cultural e sua aplicação às consultas e cuidados de saúde das pessoas que se identificam como lésbicas, *gays*, bissexuais transgêneros, *queers*, intersexuais (LGBTQI) ou Dois-Espíritos. Um melhor entendimento das questões de saúde da comunidade LGBTQI e Dois-Espíritos é essencial para reduzir as atuais barreiras que eles encontram no acesso aos cuidados de saúde.

**Método:**

foi desenvolvido um kit de ferramentas educacionais online que inclui jogos de simulação virtual e recursos selecionados. O processo de desenvolvimento abrangeu envolvimento comunitário, encontro para formação de equipe, desenvolvimento de desfechos de aprendizagem, mapas de pontos de decisão e escrita de roteiros para gravações. Um site-sistema de gerência da aprendizagem foi desenhado para apresentação dos objetivos de aprendizagem, recursos selecionados e os jogos virtuais.

**Resultados:**

o *Kit de Ferramentas de Enfermagem para Orientação Sexual e Identidade de Gênero* foi criado para a promoção da humildade cultural na prática de enfermagem. O kit de aprendizagem foca nos encontros que usam humildade cultural para atender às necessidades específicas das comunidades LGBTQI e Dois-Espíritos.

**Conclusão:**

nosso inovador kit de ferramentas educacionais online pode ser usado para promover o desenvolvimento profissional de enfermeiros e outros profissionais da saúde nos cuidados aos indivíduos LGBTQI e Dois-Espíritos.

## Abstract (es)

**Objetivo:**

desarrollar e implementar una herramienta educativa en línea para satisfacer una demanda en la educación de enfermería sobre el concepto de humildad cultural y su aplicación a las consultas y atención de la salud de las personas que se identifican como lesbianas, gais, bisexuales, transgénero, *queer*, intersexuales (LGBTQI) o Dos Espíritus. Una mejor comprensión de los problemas de salud de la comunidad LGBTQI y Dos Espíritus es esencial para reducir los obstáculos que enfrentan actualmente para acceder a la atención médica.

**Método:**

se desarrolló un kit de herramientas educativas en línea que incluye juegos de simulación virtual y recursos seleccionados. El proceso de desarrollo incluyó la participación de la comunidad, reuniones para la formación de equipos, desarrollo de resultados de aprendizaje, mapas de puntos de decisión y redacción de guiones para grabaciones. Se diseñó un sitio-sistema de gestión del aprendizaje para presentar los objetivos del aprendizaje, los recursos seleccionados y los juegos virtuales.

**Resultados:**

se creó el *Kit de Herramientas de Enfermería para la Orientación Sexual y la Identidad de Género* para promover la humildad cultural en la práctica de la enfermería. El kit de aprendizaje se centra en reuniones que utilizan la humildad cultural para satisfacer las necesidades específicas de las comunidades LGBTQI y Dos Espíritus.

**Conclusión:**

nuestro innovador kit de herramientas educativas en línea se puede utilizar para promover el desarrollo profesional de enfermeros y otros profesionales de la salud para el cuidado de personas LGBTQI y de Dos Espíritus.

## Introduction

LGBTQI2S (lesbian, gay, bisexual, transgender, queer, intersex and two-spirit) individuals remain one of the largest underserved populations in healthcare and continue to experience significant barriers and health disparities^(1-4)^. The lack of culturally sensitive and knowledgeable healthcare providers has been identified as a primary contributor to these disparities^(5-7)^. The lack of knowledge can be related to minimal training on LBGTQI2S health and wellness in formal education of nurses^(8-10)^, physicians^(11-12)^ and social workers^(13-14)^. This lack of educational preparedness indicates a substantial lack of awareness of LGBTQI2S health issues and suggests a need to develop and incorporate LBGTQI2S content into healthcare practitioner curriculum.

Nursing faculty have noted the challenges of including LGBTQI2S content into nursing programs^(8,15)^. Barriers impacting faculty inclusion of content includes concerns related to their readiness^(8-9)^, their ability to teach LGBTQI2S content^(16-17)^, and the availability of evidencebased, up-to-date teaching resources^(17-18)^. Despite these barriers to inclusion in the nursing curriculum, a study by Sirota^(18)^ found that the majority of nursing faculty felt that teaching LGBTQI2S health content was important.

Cultural competence and cultural humility are approaches required in nursing curricula as they are essential for practitioners in creating ongoing cultural safety with LGBTQI2S patients^(19-21)^. Cultural competence refers to developing knowledge, attitudes, and skills in effective cross-cultural work^(22)^. Cultural humility is a culture care framework for practitioners to develop collaborative patient relationships. It was developed in response to the limited non-intersectional nature of cultural competence, expanding the definition of culture to include processes other than just ethnicity and nationality^(23)^. Cultural humility utilizes critical selfreflection, mutual trust grounded in authentic relationship building, and identifying, understanding, and mitigating individual and systemic power imbalances^(24-27)^. Some now collapse these features into the evolution of cultural competency^(28-29)^ or recognize cultural humility as a process and cultural competency as a product^(30-31)^. Critically, however, pairing cultural humility and cultural competency in this way centres the practitioner and excludes the patient. Thus, using cultural competencies in a humble manner in collaboration with patients producing cultural safety is an improved outcome^(20)^. Cultural safety moves away from looking at differences and instead focuses on holistically and collaboratively ameliorating the colonial power differentials produced in the healthcare system by those differences^(32)^. These concepts are essential in creating decolonialized healthcare spaces that allow for the individual telling of LGBTQI2S stories and removal of the reliance of healthcare providers on stereotyped, Euro-centric narratives and contexts^(33)^.

**Figure 1:**
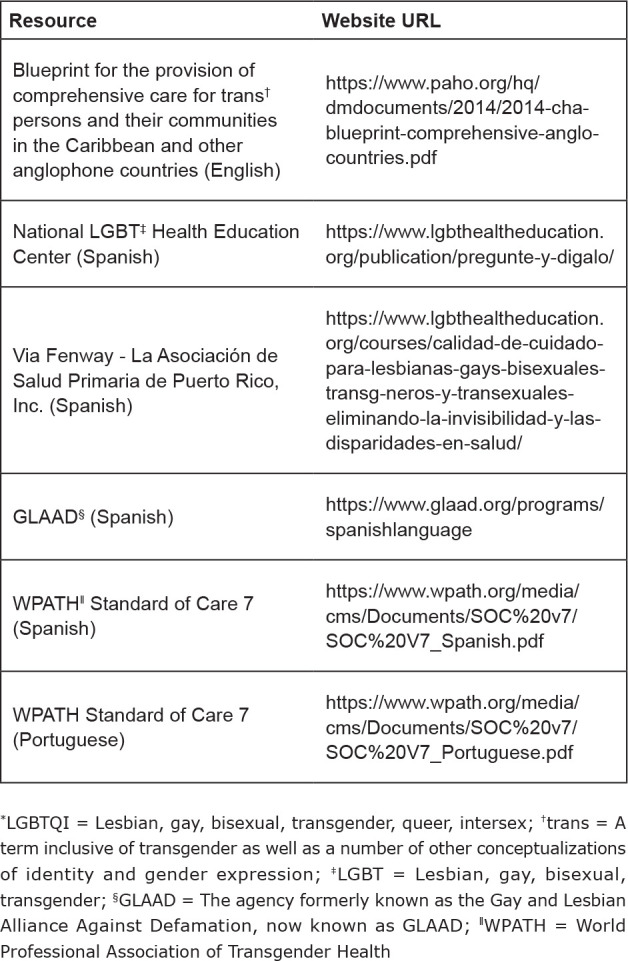
Sample of International LGBTQI^*^ and Two Spirit Resources

In Canada, a bilingual country, we have access to some LGBTQI2S resources available in French, but minimal resources are available in other languages. Outside of Canada, resources in other languages were identified (See Figure 1); however, we may be unaware of additional national, regional, or clinical guidelines.

The objective is to develop and implement an online education resources to address a gap in nursing education regarding the concept of cultural humility and its application to healthcare encounters with persons who identify as lesbian, gay, bisexual, transgender, queer, intersex (LGBTQI) or Two-Spirit. Improved understanding of LGBTQI and Two-Spirit community health issues is essential to reducing the healthcare access barriers they currently face.

## Method

The Canadian Institutes of Health Research (CIHR) - Institute of Gender and Health created a knowledge translation award for graduate student trainees with the theme of *Innovative Thinking to Support LGBTQI2S Health and Wellness* ^(34)^ *.* Design thinking methodology was used to create a solution to address nurses’ lack of knowledge about LGBTQI2S healthcare issues^(35-36)^. Design thinking is a product development methodology that emphasizes creativity, interdisciplinarity, and innovation^(35)^. Graduate students from across Canada (including EZ, BC and CS) gathered at a design jam event where they partnered with local healthcare providers, LGBTQI2S community members and marketing experts. In the three-day-long event, they consulted with community stakeholders and defined a key knowledge to action gap: *Educating nurses to use cultural humility to improve LGBTQI2S individual’s interactions in healthcare*. In collaboration with LGBTQI2S health leaders and marketing experts, they then brainstormed solutions for the problem, prototyped a product, and planned the user experience of an online educational toolkit for nurses and nurse educators to address the knowledge translation gap. The bulk of the knowledge translation award was then used to develop the educational website. In keeping with the LGBTQI2S community member involvement at the CIHR design jam event, the website development team chose to integrate LGBTQI2S community knowledge users and stakeholders throughout the development process, creating an integrated knowledge translation toolkit. Integrated knowledge translation collaboratively includes knowledge users and community stakeholders throughout the research and development processes^(37-38)^.

### Team Building

At the onset, we brought the team of simulation design experts and LGBTQI2S content experts together with four community stakeholders in a face-to-face visioning meeting. We reviewed the objectives and discussed how a framework of cultural humility would guide the educational toolkit’s content and development. Content experts shared some of the main challenges faced by LGBTQI2S individuals when accessing health care, which was a large and integral portion of the toolkit’s development. As the toolkit was designed to be representative of a large diversity of community members, there were numerous fears and concerns about tokenism, inadvertently furthering certain stereotypes, and being able to truly represent an authentic experience from a realistic vantage point of the LGBTQI2S individual. Together the group determined that four virtual simulation games (VSG) and two to three mini-games would be created for the toolkit.

The priority scenario topics identified were: 1) an older gay man experiencing grief following the loss of his partner; 2) a transgender youth experiencing significant anxiety; 3) a lesbian women experiencing pregnancy assumption; and 4) a transgender man experiencing misnaming. There were many topics that were identified and could be addressed with additional time and resources. The group chose to limit the number of scenarios to ones that all agreed were the highest priority and most feasible. The team proposed other mini-games that could be produced if resources allowed. Several additional issues were explored within these scenarios, including coming out to the healthcare provider, assessing suicide risk, and creating safer clinic spaces. Addressed healthcare provider learning needs included communication, heterosexism, not reinforcing stereotypes, creating safe spaces, confidentiality, and asking good questions.

### Virtual simulation game design

To design the games, the team broke into four writing groups with at least one simulation expert and one content expert *per* group. Additional community stakeholders, from organizations such as SAGE Advocacy & Services for LGBT Elders, and Rainbow Health Ontario, with specific lived experience, were invited to participate. Writing sessions were conducted online using Zoom video conferencing, with the researchers leading each team through the VSG design process previously developed^(39)^. This process consisted of first determining specific learning outcomes for each VSG, creating a learning outcomes assessment rubric with levelled descriptors, and creating a decision point map with three responses and rationale *per* decision point.

Following peer review by the team, the writing teams developed the filming scripts, which outlined the setting, roles, actions, dialogue, emotions, and nonverbal communication which actors would portray in each video clip. The writing groups made a conscious effort to ensure scripts were representative of an entire range of experiences. There was great care taken to reduce any possible stereotypes in the development of the scripts; however, the development team acknowledges they may persist. Filming scripts were peer-reviewed prior to filming by community members with lived experience to ensure authenticity. Once scripts were reviewed and approved, they were sent for translation into French language versions and reviewed by Francophone LGBTQI2S community members for slang and dialect authenticity.

Filming of each game took place at a university clinical education centre over approximately four hours *per* scenario. The recording teams used a head-mounted GoPro camera and an iPad to control and view the video recordings to offer a first-person perspective of the nurse during the nurse-patient encounter. Authentic members of the LGBTQI2S community were recruited as actors to play the patient roles wherever possible, and team members took on the nursing roles. The groups encountered difficulties getting authentic actors who were comfortable and confident enough to represent the roles. This was primarily due to roles representing a minority group and documenting a minority experience. Many people did not feel comfortable globally representing those groups on film due to concerns about visibility and fears of repercussions on the actor’s personal lives after being filmed.

Live actor filming was essential; however, because the actors are putting themselves out there as visibly representative of an oppressed group for filming, they had reservations concerning how the experience could affect their lives outside of the experience. More than one actor recruited, subsequently dropped out before filming occurred, thus delaying filming while the creators had to search out another available actor, which may not have been representative of the person being portrayed. Additionally, and partially due to our team’s English only members, there were difficulties getting actors who were both representative of the minority group who were fluent in a second language (French), thus compounding the issues of being “out on film”. Further, even though French is an official language and prominent in our region, it is still a minority population. The intersectionality of being part of both the LGBTQI2S and Francophone communities meant that a very small population of people was being asked to take on a public role.

### Website and Course Design

The toolkit required the inclusion of a variety of curated educational resources. The development team and content experts determined industry and academic resources (i.e. journal articles, web-based publications, government reports and community support sites) to enhance learners’ knowledge and competency. Resources were aligned to meet program and script learning outcomes and mapped against developed rubrics.

To make the toolkit available to the broadest possible audience, the development team decided to ensure all resources would be available online with a distinct web presence. The team selected www.soginursing.ca as the toolkit domain. A critical requirement of the website was to enable the curated educational resources to be entirely searchable through an online database as well as directly linked from the course rubrics and scenarios. The website is contained within a WordPress content system, which allows the team to provide multiple people with access to update the resources regularly within a userfriendly interface. Editing of the resources can be done independently of updates to the site and VSGs.

While the website housed all the resources and links to the program and scenarios, the program itself was determined to be best hosted off the website, on a simulation repository focused on nursing education. The repository provided an already established Learning Management System (LMS) as well as an existing audience of nursing educators who were already using VSGs in their teaching.

Within the LMS, existing VSGs are housed within their own “course”, which includes all of the components required to run the VSG within an educational setting. Courses within the repository follow best practices in online pedagogy, including critical and individual learning outcomes, assessment rubrics and debriefing processes. The toolkit VSGs a single course within the LMS, which enables learners to move linearly throughout the scenarios and complete personal bias quizzes and rubrics. Each scenario is equipped with an interactive rubric that allows learners to rate their own experiences and perceptions before and after the scenario. Based on their selections in the rubric, learners are provided with a list of resources to improve in areas where they rated themselves poorly.

While the primary website is entirely in English, a second complete French mirror is currently in translation. This will enable the team to capture and store French language resources, which can be linked to a French version of the VSG course within the repository.

### Usability and Evaluation Testing

The website and virtual simulation games have undergone formal usability testing by a group of nursing students and faculty members from three sites to evaluate ease of use, engagement, and usefulness of the toolkit. Additionally, informal feedback has been received from users following the launch of the open-access website. Obtained feedback will inform further changes to the tools prior to implementation of a larger evaluation study with a larger multi-site sample of nursing students and nurse practitioner students. Ethics approval has been obtained, and further funding has been secured to support this evaluation phase. **Results**

Our objective was to address an identified gap in nursing education through the development of a cultural humility toolkit and its application to healthcare encounters with the LGBTQI2S community. We generated an easy-touse, comprehensive online LGBTQI2S Cultural Humility Toolkit available in both English and French languages. Our main goal of this toolkit is to enhance healthcare providers’ cultural humility through self-reflection, knowledge acquisition, and knowledge application. Resources are guided by key learning outcomes based on the prioritized health needs of members of the LGBTQI2S community.

The critical concepts and terms within the toolkit are reinforced through introductory audio presentations hosted by content experts. The narrated, multimedia presentations explore core concepts of cultural humility and cultural safety, sexual orientation, and gender identity (SOGI) terminology and discuss the unique generational health assessment needs of this diverse population.

The core content of the toolkit includes four bilingual virtual simulation games. The program and scripting learning outcomes guided the development of each game and respective resources. The first VSG focuses on an interaction between a healthcare provider and an older gay man grieving after the death of their life-partner. Through the first VSG, learners explore principles including: personal assumptions about heterosexism, applying principles of cultural humility when asking health assessment questions, ensuring the patient’s current health needs are addressed and prioritized, and implementing prioritized holistic nursing interventions to promote safety recovery and improve client health and well-being. The second VSG explores the urgent assessment of a transgender youth experiencing a situational crisis. Learning outcomes focus on creating a safe space to facilitate communication, applying cultural humility when completing a HEADSS assessment, and exploring sexual health practices to identify risk (pregnancy, sexually transmitted diseases, and relational safety). The third VSG takes place in a woman’s clinic where a nurse practitioner completes an annual examination for a queer young adult. This VSG explores issues of heterosexism and gender bias and applies principles of cultural humility when obtaining a sexual health history. The final VSG involves an interaction between a healthcare provider and a transgender person seeking assessment for acute medical concerns. Learning outcomes focus on the impact of cultural assumptions, including misgendering, establishing supportive interactions, and performing a gender-identity sensitive health history.

In addition, the toolkit also includes three short vignette mini-games targeting common issues faced by non-heterosexual and non-cisgender people when entering the healthcare environment. Current mini-games address: 1) heteronormative assumptions by portraying a same-sex couple and their child accessing health care; 2) gender assumptions when a transgender person’s legal gender information differs from their lived gender and 3) inappropriate disclosure of personal information between health care professionals. Future mini-games will include bisexual invisibility when assessing health risk behaviours, decision making for parents of an intersex infant, and contact tracing, consent, and disclosure regarding men who sleep with men but otherwise identifies as heterosexual.

Each VSG has embedded self-assessment rubrics to support reflective practice. Based on the script learning outcomes and related indicators, learners self-assess their perceived competency (novice to competent learner).

When learners identify a potential gap in knowledge, they are directed to learning resources within the online database on the website. The website further allows learners to search associated resources based on keywords and related categories.

Based on International Nursing Association for Clinical Simulation and Learning *Standards of Best Practice* ^(40)^, the toolkit provides options for virtual simulation debriefing. Learners can engage in self-debriefing by completing selfassessment rubrics and responding to the VSGs reflective questions available on the website. These reflective questions were designed to examine personal values, beliefs, and biases further. The toolkit also provides information about other optional debriefing methods such as asynchronous, synchronous, and in-person debriefing when the VSGs are used within a nursing program.

Informal feedback received from users following the launch of the open-access website informed several modifications to the toolkit. For example, it was noted that images used on the website, while reflective of gender diversity but lacked visible representation of people of colour. We responded immediately to this feedback by adding additional photos inclusive of more ethnic diverse individuals, as well as individuals with physical disabilities.

## Discussion

We were successful in meeting our objective and goals for this educational intervention. The development of the SOGI nursing online educational toolkit brought together a group of LGBTQI2S subject matter experts, educators, simulation experts and an e-learning specialist, creating a unique interprofessional collaboration^(41)^. Key priority was ensuring the authenticity of the patient experience in each VSG. Inviting LGBTQI2S individuals and members of community organizations to the VSG development sessions allowed us to capture realistic patient experiences.

The value of using authentic gender minority individuals as standardized patients has been recognized in medical education, however there are challenges to recruitment and ensuring psychological safety^(42)^. Cultural humility principals were used to ensure the psychological safety of actors, which included self-awareness of our own strengths and limitations, openness to exploring new ideas, being egoless, providing supportive interactions, and engaging in self-reflection and critique^(43)^.

One of the biggest challenges was our inability to integrate bisexual, intersex, and two-spirit content into the online educational toolkit. Despite wanting to ensure that we captured the patient experiences of all LGBTQI2S identities, we were unable to network with appropriate community members and organizations to ensure cultural safety. In an attempt to ensure an authentic patient experience, we wanted to approach these topics more thoughtfully.

The online games are available in both official Canadian languages, English, and French. However, many of the curated resources are only in English. We anticipate some resources in French will be difficult to find or may not exist. The complete translation of the overall website to French is still forthcoming. Similarly, Two-Spirit content is still forthcoming as we were unable to network properly with the Indigenous community to ensure the cultural safety of the proposed content.

Nursing research related to LGBTQ health has shown slow growth from 2009 to 2017, and much of this research focuses on lesbian and gay populations with little attention given to transgender and gendernonconforming populations^(44)^. Additionally, there are few published studies that have evaluated the effectiveness of educational interventions aimed at improving knowledge and attitudes regarding LGBTQ care^(44)^. An educational intervention for nursing students consisted of three online modules and an in-class simulation exercise aimed and resulted in significantly improved cultural competence scores related to care of LGBT persons^(45)^. Another study evaluating a lecture-based educational intervention entitled “LGBTQ Cultural Competence for Registered Nurses” demonstrated significantly improved knowledge but not attitudes toward LBGTQ people^(44)^. Simulation scenarios have previously been created on the topic of gender minorities for use in nursing education. Anecdotal results from one project using standardized patient simulations indicated this approach supported knowledge acquisition on the needs of the transgender and gender nonconforming patients, and learners felt they could transfer the skills attained to the clinical setting^(46)^. We anticipate that evaluation of our educational toolkit will further support these findings.

An important aspect of our VSGs was role-modeling of culturally humble communication and behaviours during health care encounters. For example, the importance of pronoun-checking was validated in a study conducted with LGBTQ youth^(47)^, thus, the nurses in our VSGs demonstrated how to ask about pronouns and provide their own pronouns to the patient. Our VSGs also addressed health care topics that are important for primary care providers including nurse practitioners interacting with LGBTQ patients when conducting a sexual health history and screening for intimate partner violence^(48)^.

### Next Steps

A multi-phase, multi-site, mixed-methods study of the feasibility, usability and learning outcomes associated with completing the SOGI-Nursing eLearning toolkit is currently underway. Phase 1 involves usability testing of the website; participants will be undergraduate and graduate nursing students as well as nursing faculty. We will also recruit 5-10 LGBTQI2S content experts to review the website and the VSGs to provide feedback about the content. Phase 2 of the study will explore the implementation and evaluation of the SOGI-Nursing online educational toolkit. Further plans may include evaluating the learning outcomes and their usefulness in knowledge, attitude, and behaviour change.

Plans for additional VSGs are in development and include 1) a bisexual woman engaging in risky health behaviours and 2) parents of an intersex child engaging with a nurse, 3) contact tracing of a man who has sex with men who has a reportable sexually transmitted infection. We acknowledge the need to include an appropriate TwoSpirit perspective and therefore continue to search for collaborative opportunities with the Two-Spirit community.

The application of the toolkit is multifaceted and relevant for a variety of health disciplines. Within undergraduate education programs, the toolkit can support the development of learner’s cultural humility, professional knowledge, skills, and attitudes. Individual VSGs can be used as stand-alone games or as -simulation preparation prior to a live standardized patient encounter. Not restricted to the lab environment, when implemented in theory courses and guided by the educator, critical thinking questions within each VSG can stimulate intentional group discussion. For practicing healthcare providers, the toolkit is ideal as a professional development activity that is required by many professional regulating organizations. While still only available in English and French, the online educational toolkit provides a range of resources for helping nurses and nurse educators gain knowledge in providing care to LGBTQI2S populations.

To enhance global use of the toolkit, we are exploring the opportunities to expand and collaborate with subject matter experts and educators in other regions, such as South America, Europe, and Asia. There is a potential for translation/overdubbing of the VSGs into other languages such as Spanish or Portuguese. There is also the potential to collaborate with other groups to develop scenarios that best reflect their relevant cultural, religious, and social circumstances, such as South America’s indigenous populations.

## Conclusion

Despite increased awareness surrounding LGBTQI2S populations and the unique health disparities affecting these individuals, nurses continue to lack educational preparedness regarding caring for this population. The development of the SOGI Nursing website is the first step to address this gap. Our innovative educational learning toolkit can be used in academic and healthcare settings to provide professional development to better prepare the next generation of nurses and other healthcare practitioners to care for individuals who identify as LGBTQI2S.
